# The development of an “Engagement in Physical Education” scale

**DOI:** 10.3389/fspor.2024.1460267

**Published:** 2024-11-27

**Authors:** Andy Stringfellow, Chih-hsuan Wang, Claudio F. G. Farias, Peter A. Hastie

**Affiliations:** ^1^ Department of Curriculum, Instruction and Foundations, Marshall University, Huntington, WV, United States; ^2^ Department of Educational Foundations, Leadership and Technology, Auburn University, Auburn, AL, United States; ^3^ Faculty of Sport, University of Porto, Porto, Portugal; ^4^ School of Kinesiology, Auburn University, Auburn, AL, United States

**Keywords:** participation, involvement, survey, CFA, physical education

## Abstract

**Purpose:**

The purpose of this paper is to present a scale that assesses students' perceptions of their engagement in physical education. The scale assesses all four dimensions of engagement (agentic, cognitive, behavioral, and emotional) in order to be consistent with the contemporary notion of engagement used in current educational research.

**Method:**

A total of 231 eighth and ninth-grade students (108 boys, Mage = 14.55) completed a 21-item scale, with items taken from previously validated scales, but with an added string relating to physical education lessons. Following model respecification by the examination of standardized residual covariances to remove items, the factor loading for each of the items on the scale was examined.

**Results:**

The results from a confirmatory factor analysis showed that there was psychometric support for an 18-item survey in which each of the four *a priori* latent variables was kept in the validation of the four-factor hypothesized model. [*χ*^2^ = 226.01, df = 125, *χ*^2^/df = 1.808, GFI = .903, RMSEA (90% CI) = .061 (.048–.073)].

**Conclusion:**

Given the research on engagement within physical education has been beleaguered by the lack of a robust operational definition, this scale allows for the comprehensive measurement of the different components of engagement.

## 1 Introduction

Student engagement plays an important role in shaping educational achievement at both macro (1) (retention and participation in school activities), and micro (2) (performance and learning) levels. By consequence, the concept of “engagement” has been a frequent topic within educational research in general, and in physical education specifically. Indeed, in the sport pedagogy literature, one can find over 50 studies incorporating the term in their titles.

For the purposes of this paper, we follow the definition of engagement as an active, effortful, goal-directed interaction with one's learning environment (3). However, within the research on physical education, there has been considerable diversity in the way that engagement has been applied (4). Sample expressions have included “effort” (5), “persistence” (6), “active participation” (7, 8), “on-task behavior” (9), and have used being “physically active” (10) as a proxy measure. To further muddy the waters, it should be noted that only 50% of papers in physical education provide an actual definition of engagement itself, and only slightly over half (56%) use instruments to measure students' engagement (4).

While engagement is a key construct in motivational models because it is considered a primary pathway by which motivational processes contribute to learning and development (11), it is important not to equate engagement with motivation. More exactly, students' motivation might better be seen as a precursor to engagement as motivation is insufficient for one to be engaged. That is, the main reason students show strong classroom engagement is that they initially experience a fulfillment of engagement-boosting psychological needs (12).

As it is teachers who largely shape a supportive motivational climate, many scholars have examined what specific teaching styles best achieve this goal. In the main, these studies and subsequent interventions are grounded in Self-Determination Theory (13) wherein it is proposed that all people have three essential, inherent psychological needs. These are autonomy, competence, and relatedness. Recently, a panel of experts on SDT have identified 57 motivational behaviors that teachers can use that provide autonomy, competence and relatedness support to students (14).

Concurrent with this narrowing of the lens to focus on motivation and engagement has been an expansion on the multi-dimensional conception of engagement. Originally, considered two dimensional (behavioral and emotional), then three (affective, behavioral, and cognitive), the most contemporary approach is the four-dimensional framework that is widely considered today. This four-dimensional perspective on engagement adds the idea that in addition to their behavioral, emotional, and cognitive engagement, some students become so deeply involved in lessons that they initiate actions that support their learning, and at times, contribute to the evolution and growth of the lesson. This concept was given the label of “agentic engagement” by Reeve and Tseng (15) who suggested that the strategies most used by students to proactively and intentionally contribute to their learning include expressing their preferences, asking questions, and letting the teacher know what they like, need, and want. Zambrano et al. (16) have found that students have three rationales for agentically engaging. These are to prompt teacher support, support personal experiences of motivation and learning, and to create a more desirable learning environment for the classroom community.

While it was noted earlier in the paper that some research in physical education engagement has incorporated formal measures of the construct, it should be pointed out that these instruments are limited in one of two ways. First, several studies used items or subscales developed in classroom settings and applied them directly to physical education (17, 18). Second, those who borrowed items from previously validated research in physical education and included acceptable factorial validity confirmatory factor analysis (8, 9) did not include items relating to students' agentic engagement. That is, these surveys focused exclusively on the behavioral, cognitive, and emotional dimensions of engagement, and did not include items that measured the extent to which students aim to create motivationally supportive learning environments for themselves.

As a result of the limitations of the previous measures of engagement within physical education, the objective of this study was to produce a survey that included valid items relating to agentic, cognitive, behavioral, and emotional engagement, and which had absolute fit indices showing a good fit for this model. It was considered that given the multidimensional nature of engagement, a self-report measure could provide the most accurate insight into an individual's internal thoughts and feelings about their involvement in specific contexts. While observation measures can provide some report of an individual's behavioral and/or agentic engagement, it does not allow for understanding the subjective experience of participants in the cognitive and emotional dimensions.

## 2 Methods

### 2.1 Construction of the item pool

To develop the “Engagement in Physical Education Scale,” items from previously validated scales were used with an added string relating to physical education lessons. The final draft scale consisted of 21 items with four subscales. The subscales included agentic, cognitive, behavioral, and emotional engagement. All of the items were scored using an ordinal five-point Likert scale that ranged from “strongly disagree” to “strongly agree” with an answer of “neutral” as the midpoint.

#### 2.1.1 Agentic subscale

Seven items from two different previously validated scales were adapted to provide items for the agentic subscale (see Table 1). Most items were taken from the Hit-Steer Observation System (19, 20). The Hit-Steer Observation system was used to assess classroom behavior by counting the number of times a student tries to impact the teacher (a “hit”) and if the student's actions changed the teacher's behavior (a “steer”). Items two through four were initially used by Reeve and Tseng (15). Reeve and Tseng created this subscale to determine if agentic engagement had a positive correlation with behavioral, cognitive, and emotional engagement. These items showed moderate to high positive correlations with the other three aspects of engagement.

**Table 1:** Original statement, source, and modifications of survey items.

	#	Original statement	Source	Modified statement
Agentic	1	I let my teacher know what I need and want.	1	In PE, I let my teacher know what I need and want.
	2	I let my teacher know what I am interested in.	2	In PE, I let my teacher know what I am interested in.
	3	During class, I express my preferences and opinions.	2	During PE, I express my preferences and opinions.
	4	During class, I ask questions.	1	During PE, I ask questions so I can learn.
	5	When I need something in this class, I will ask the teacher for it.	1	When I need something in PE, I will ask the teacher for it.
	6	I adjust whatever we are learning so I can learn as much as possible.	1	In PE, I change whatever we are learning so I can learn as much as possible.
	7	I try to make whatever we are learning as interesting as possible.	1	In PE, I try to make whatever we are learning as interesting as possible.
Cognitive	8	When I study, I try to connect what I am learning with my own experiences.	2	When I practice skills for PE, I try to connect what I am learning with my own experiences.
	9	I try to make all the different ideas fit together and make sense when I study.	2	I try to understand why I practice skills for PE.
	10	When doing schoolwork, I try to relate what I’m learning to what I already know.	2	When participating in PE, I try to relate what I’m learning to what I already know.
	11	I make up my own examples to help me understand the important concepts I study.	2	I practice on my own to help me understand the important concepts taught in PE.
Behavioral	12	I listen carefully in class.	2	When I’m in PE, I listen carefully.
	13	I pay attention in class.	2	I pay attention in PE.
	14	I try very hard in school.	2	I try hard to do well in PE.
	15	I work hard when we start something new in class.	2	In PE, I work as hard as I can.
	16	I participate in class discussions.	2	When I’m in PE, I participate in PE activities
Emotional	17	When we work on something in class, I feel interested.	2	When we work on something in PE, I feel interested.
	18	Class is fun.	2	PE is fun.
	19	I enjoy learning new things in class.	2	I enjoy learning new things in PE.
	20	When I’m in class, I feel good.	2	When I’m in PE, I feel good.
	21	When I am in class, I feel curious about what we are learning.	2	When we work on something in PE, I get involved.

1. Reeve (2013), 2. Reeve & Tseng (2011).

The construction of the scale by Reeve and Tseng (15) was based mainly on self-determination theory (SDT) (21) and all of the major theories of student motivation. Items one and five were new candidate items designed to assess the student's contribution to the learning environment (20). Items six and seven were also new candidate items designed to assess the student's contribution to their learning. In the previous study, items one through five loaded on the agentic engagement factor with strong positive correlations. Items six and seven did not load on the agentic factor. However, the items did load on cognitive engagement with strong negative correlations (21). The present study included items six and seven for use in the engagement in physical education scale. The addition of the wording “in PE” to items six and seven was the justification for using these items in the new scale with the hope that the addition of the context-specific wording would have an impact on the factor loading.

#### 2.1.2 Cognitive subscale

To assess cognitive engagement, four statements from Reeve and Tseng's (14) cognitive subscale were used. These were adapted by adding the wording “in PE” to make the statements specific to physical education. The four items used are shown in Table 1 and are represented by items eight through 11 in the present survey.

Reeve and Tseng's cognitive subscale was based on the adopted cognitive items on Wolters' (22) Learning Strategies Questionnaire. Wolters' questionnaire was based on the subscale for cognitive strategies of the Motivated Strategies for Learning Questionnaire (23). Wolters' objective was to explore the association between achievement goal theory (24, 25, 26, 27) and student motivation, cognitive engagement, and academic achievement. The four items selected from the original subscale were designed to assess the sophisticated learning strategies of college students Reeve and Tseng's work (15) demonstrated an internal reliability of *α* = .82. By selecting these items for the newly developed scale and adding the wording “in PE,” the hope was that these items would show greater internal consistency because it is context specific.

#### 2.1.3 Behavioral subscale

To assess behavioral engagement, the statements from Reeve and Tseng's (15) Questionnaire to Assess Four Aspects of Engagement behavioral subscale were used. The survey of Reeve and Tsang was developed using Miserandino's (28) task involvement questionnaire. Miserandino's questionnaire was specifically designed to measure the perceived behavioral engagement of students in the classroom. Four of the items loaded on attentiveness and one loaded on participating. Three items loaded with a questionable level of internal consistency. The current survey item 12 is the same as item one in Miserandino's task involvement questionnaire, item 14 is reflective of item four, and item 16 is the same as item 28.

Two of the items loaded with weak internal consistency. The current survey's item 13 is the same as item five in Miserandino's (28) task involvement questionnaire which showed *α* = .54, and item 15 reflects item four in Miserandino's task involvement questionnaire which showed *α* = .59. All five items presented by Reeve and Tseng (15) (internal reliability *α* = .82) were adapted by adding “in PE” to make the statements specific to the physical education context for the current survey. These five items are represented in the present survey as items 12 through 16 in Table 1. By adding context-specific wording, the statements can represent student self-perceptions of attentiveness, participation, and effort in a physical education class.

#### 2.1.4 Emotional subscale

To assess emotional engagement, statements based on Reeve and Tseng's (15) emotional subscale were used. The current survey contains items 17–21 in Table 1. These items were adapted from Reeve and Tseng by adding the wording “in PE” to make the statements specific to physical education. These items showed an acceptable level of internal consistency in the previous study by Reeve and Tseng (*α* = .78).

### 2.2 Participants

Criterion-based sampling was used to select the participants. Participants included 231 eighth and ninth-grade students (108 boys 46.75%, 115 girls 49.78%, eight unreported 3.46%) aged between 12 and 17 years (Mage = 14.55, SD = 2.65) enrolled in a mandatory physical education class. One hundred and two eighth-grade (44.16%) and 129 ninth-grade students (55.84%) participated in the initial administering of the survey. The eighth grade level was chosen as it represents the upper level of middle school or junior high school, whereas ninth grade (at least in the United States) is often the last year when students will take a required physical education course. By consequence the eighth and ninth grades represent the largest target group of students participating in physical education.

The justification for the sample size for this research was based on the information provided by previous researchers in scale validation (29, 30). Most of these recommendations state that a minimum ratio of 10 participants for every item on the scale is needed; while a higher ratio of participants to items is desirable, studies that have been completed with a lesser ratio were also reported. The present study's goal was to include at least 10 participants per item.

### 2.3 Procedures

After receiving approval from the school boards and IRB, individual schools and physical education, teachers were asked to allow the research to take place. Participants were then provided a parental/guardian consent/assent form that was completed and returned for them to be given access to the survey.

The anonymous survey was completed during their regular physical education classes, and students were asked not to place any identifying marks on the survey. The teacher(s) of the classes did not have access to the names of the students who chose to participate or not. The teachers were also not given any access to the collected data.

### 2.4 Data analysis

The IBM SPSS v.24 was used for data screening for outliers. Eleven outliers were discovered and eliminated. This was accomplished by running a regression analysis to test Mahalanobis distance and then using the explore function to test outliers for Mahalanobis chi-square. This calculation generated a boxplot that clearly showed the outliers. Eliminating the outliers brought the sample size to 220 students (123 boys 55.91%, 92 girls 41.82%, five unreported 2.27%) aged between 12 and 17 years (Mage = 14.54, SD = 2.85). Ninety-nine eighth-grade students (45%) and 121 from the ninth grade (55%) were included in the final analysis.

To establish construct validity, confirmatory factor analysis (CFA) was applied to the data to examine the structural features of the model using SPSS AMOS v.24. Analysis property outputs included standardized estimates, residual moments, and modification indices with a threshold for modification indices set at a value of four.

The absolute fit measures examined in this study included chi-square divided by the degrees of freedom (CMIN/DF). An obtained value of less than two implies a good fit (31) while an obtained value less than three is considered acceptable (31). A goodness of fit index (GFI) was also calculated. GFI is similar to R2 and produces values between zero and one with one being a perfect fit. Obtained values between .90 and .95 are acceptable; however, values larger or equal to .95 indicate a good fit (31). Third, the root mean square error of approximation (RMSEA) was determined. In this case, the value should be smaller or equal to .07, with smaller values indicating a better fit (31, 32). Next is the standardized root mean square residual (SRMSR). To have a good fit, the value should be smaller or equal to .08 (31, 32).

The relative fit indices examined for this study include the Comparative Fit Index (CFI) and the Normed Fit Index (NFI). These relative fit indices values represent where on a continuum from worst fit to a perfect fit the model lands with values ≥ .95 suggests a good fit and values between .90 and .95 as acceptable (31). The CFI is usually the fit statistic used for structural equation modeling (31, 33).

Based on the values of the fit indices produced, the table for the default model covariances was examined. The error variable with the most significant modification index (MI) value was identified, and a correlation path was drawn between the error variables associated with that MI value. This process was repeated until no more MI values produced exceeded a value of 10. These correlation paths were drawn one at a time beginning with the most substantial value, and then the data were recalculated, and the fit indices were inspected again.

After all the correlation paths were drawn, and calculations were rerun, the fit indices were again examined for model fit. Based on the standardized residual co-variances table found in the estimates matrices, items that created excessive discrepancies between the proposed model and the estimated model were considered for deletion. The identified items with values that exceeded 2.58 in the table were deleted one at a time starting with the factor with the most substantial value. No items exceeded a value of 2.58, and this method did not yield any items that could be considered for deletion.

After all justified correlation paths were drawn and all items with a standardized residual co-variance absolute value of 2.58 were eliminated, not all fit indices reached an acceptable threshold. IBM SPSS v.24 CFA was again used to produce a rotated component matrix. This matrix was inspected to determine if any factors cross-loaded onto more than one latent variable. Items that cross-loaded heavily on more than one latent variable were systematically removed one at a time. The calculations were rerun after each modification. The removed factor was added back into the model, and the next factor was removed, and the calculations were again rerun. This process was repeated for all of the heavily cross-loaded factors until the fit indices reached an acceptable level. To determine convergent and discriminant validity, the Stats Tool Package (34) was used. The table of estimates scalars correlations from the view text option of AMOS output was copied and pasted into the Stats Tool Package spreadsheet along with the estimates scalars standardized regression weights.

## 3 Results

### 3.1 Initial model evaluation

**Figure 1:**
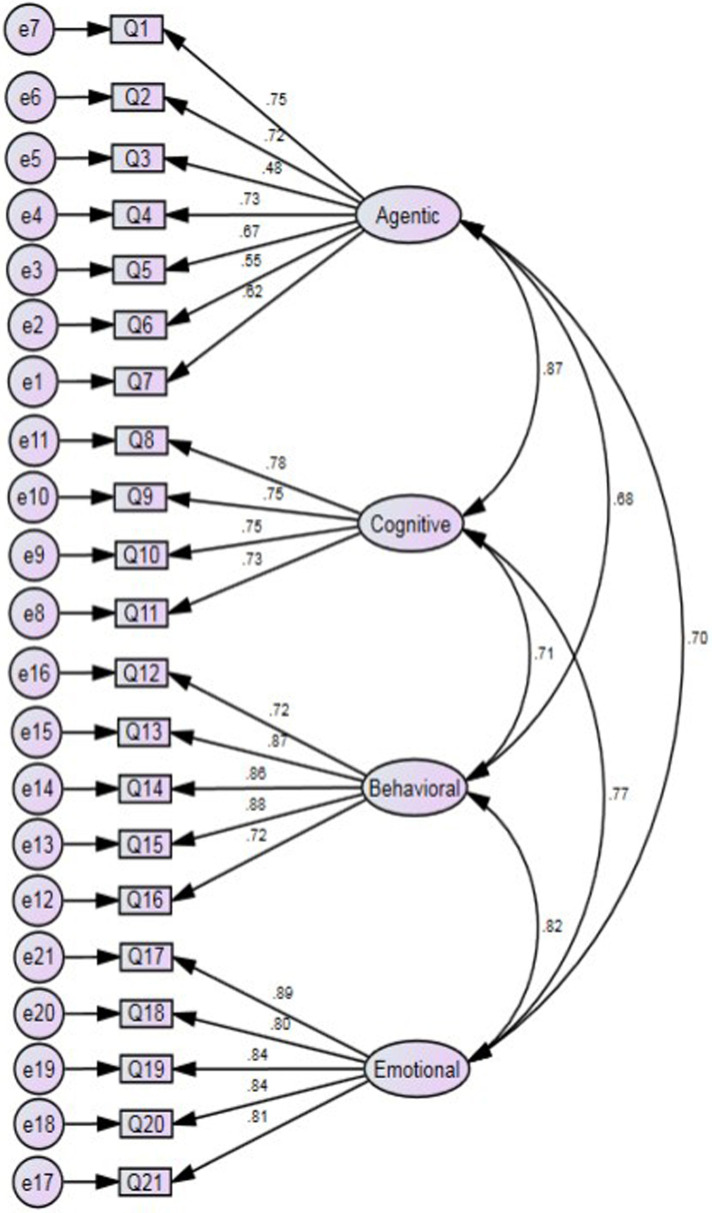
Original model with 21 items.

Evaluation of the initial model did not contain any correlations between error values. The original model contained 21 items (see Figure 1). Results from the original model evaluation indicated fit indices that revealed a statistically significant chi-square test with a value of 547.965 (df = 183), *p* < .001. Due to the large sample size, the chi-square statistic typically will show significance regardless of the other fit indices (30). Results from the initial model evaluation yielded pattern coefficients relating the factors with the items that were reasonably robust, ranging from .48 to .89. The CMIN/DF (2.994), GFI (.804), CFI (.884), NFI (.836), SRMR (.0658), and RMSEA [.094 CI (.086–.105)] taken together indicate the proposed model was on the cusp of acceptable to a good model fit. Only the absolute fit index of SRMSR (≤.10) and CMIN/DF (≤3) met the target value.

### 3.2 Modifications

The process used to improve the model consisted of drawing covariances between error variables associated with the same latent variable and having MI values exceeding 10. After each covariance arrow was drawn, the model was recalculated, and the fit indices were inspected again. New covariance arrows were drawn one at a time starting with the greatest MI value. One covariance was drawn within the behavioral engagement latent variable: e13 (associated with Q15 “In PE, I work as hard as I can”) and e14 (associated with Q14 “I try hard in PE”) were both on the behavioral variable. These items shared meaning and words that may have led to commonalities beyond shared variance, and it is reasonable to assume that these covariances would improve the model.

Three pairs of covariances were drawn within the agentic engagement latent variable: e5 (associated with Q3 “During PE, I express my preferences and opinions”) and e6 (associated with Q2 “In PE, I let my teacher know what I am interested in”); e6 (associated with Q2 “In PE, I let my teacher know what I am interested in”) and e7 (associated with Q1 “In PE, I let my teacher know what I need and want”); e3 (associated with Q3 “During PE, I express my preferences and opinions”) and e7 (associated with Q1 “In PE, I let my teacher know what I need and want”). These items shared had shared meanings of words like opinions, preferences, needs, and interests that may have led to commonalities beyond shared variance and it is reasonable to assume that these covariances would improve the model. Two pairs of covariances were drawn within the latent variable of emotional engagement: e17 (associated with Q21 “When we work on something in PE, I get involved”) and e18 (associated with Q20 “When I’m in PE, I feel good”); e17 (associated with Q21 “When we work on something in PE, I get involved”) and e20 (associated with Q18 “PE is Fun”). The commonalities of the feeling of having fun in PE, feeling good in PE and getting involved in PE seem to have warranted the covariances to help improve the model.

### 3.3 Item deletions

#### 3.3.1 Examining standardized residual covariances

At this point, the decision was made to use a more invasive approach to respecification of the model by removing some of the factors that show standardized residual covariances that exceed an absolute value of 2.58.

#### 3.3.2 CFA loadings

The next approach to improving the model was to examine the factor loading for each of the items on the scale. By inspecting the rotated component matrix, it was determined that four of the items heavily cross-loaded on more than one latent variable (Q21, Q6, Q7, and Q4). These items were removed methodically one at a time, and the model was recalculated after the removals. Fit indices were then inspected to see if the thresholds had been met. Each item was added back to the model, and the next item was removed. This process continued until all four had been removed and added back in. The next step was to remove two items at a time and then three. After Q21, Q6, and Q7 were removed together, all fit indices met the acceptable threshold, and a final version was created.

The results of the convergent and discriminant validity testing show that behavioral, cognitive, and emotional variable measures met the thresholds for composite reliability (CR), average variance extracted (AVE), and maximum shared variance (MSV) except the agentic variable. Convergent validity for agentic engagement was below .50 (AVE = .0439) and discriminant validity for agentic engagement was less than the MSV (MSV = 0.503) (see Table 2).

**Table 2:** Results of validity testing.

	CR	AVE	MSV	MaxR(H)	Behavioral	Cognitive	Agentic	Emotional
Behavioral	0.898	0.640	0.613	0.916	0.800			
Cognitive	0.806	0.580	0.546	0.807	0.729	0.762		
Agentic	0.792	0.439	0.503	0.812	0.709			
Emotional	0.917	0.733	0.613	0.919	0.783	0.739	0.695	

**Figure 2:**
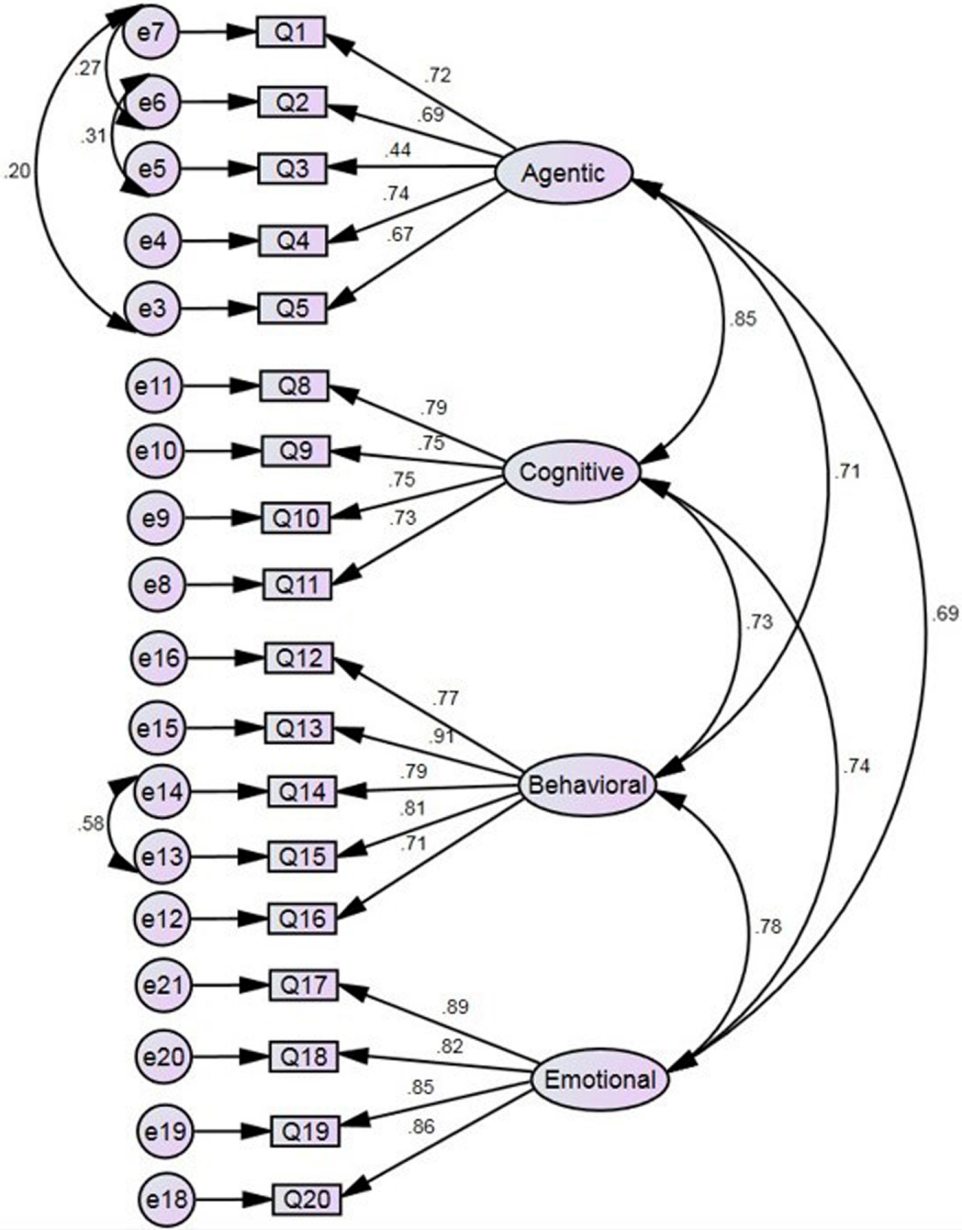
Standardized coefficients for the respecified model.

The standardized coefficients for the respecified model are presented in Figure 2. Model fit was markedly improved. The Chi-square test was statistically significant however this can be expected with large sample sizes (27), *χ*^2^ = 226.011 (125), *p* < .001, and the CMIN/DF (1.808; <2), GFI (.903; >.9), CFI (.962; ≥.9), NFI (.919; ≥.95), SRMR (.0428; ≤.08), and RMSEA (.061; ≤.07); CI (.048–.073) indicate values showing a good model fit. All of the pattern coefficients were acceptable, ranging from .44 to .91 and all were all statistically significant (all ps < .001). These results indicate that the proposed four-factor structure of the EPES was supported using the data from this independent sample. Table 3 shows the original model comparison with the proposed four-factor model fit indices.

**Table 3:** Model comparison.

Fit index	Four-factor correlated structure							RMSEA (90% CI)
	*χ* ^2^	df	*χ*^2^/df	GFI	CFI	NFI	SRMR	
Original model	547.97	183	2.994	.804	.884	.836	.0658	.095 (.086–.105)
Final model	226.01	125	1.808	.903	.962	.919	.0428	.061 (.048–.073)

## 4 Discussion

The stated goals of this study were: (a) to develop a pool of modified items from previously validated engagement scales to be specific to physical education, and (b) to use confirmatory factor analysis to verify the instrument's 21-item and four-factor internal structure of the model. The results from the CFA established the scale's validity based on its internal structure. Except for the discriminant validity values for agentic AVE (0.439) and MSV (0.503), all other values were acceptable. With these values being as close as they are, a four-factor structure showed to have acceptable model fit indices.

A questionable yet acceptable level of discriminant validity is not unprecedented. Often researchers are faced with similar results, and occasionally the decision is made to leave the model in the final form or combine two or more of the latent variables if the values are not close enough. In a recent study, the latent variables of behavior and cognitive engagement were combined to create one construct (35). It seems that this was an *a priori* decision; however, other studies have shown that the two constructs are distinctly different and can remain as a stand-alone variable (15).

In the current study, agentic engagement and cognitive engagement were highly correlated (standardized coefficient = .85). The correlation is not surprising because for an individual to experience agentic engagement, they must first be cognitively engaged (15, 21). To be cognitively engaged, one must display active self-regulation along with using complex learning strategies (15, 36, 37). Reeve and Tseng (15) based their research on agentic engagement on the Hit-Steer Observation System. One of the findings that came from this research was that students' influence attempts had a strong positive correlation with academic achievement. Moreover, self-regulation, the use of sophisticated learning strategies, and academic achievement fall into the realm of cognitive engagement. Students who actively display this type of behavior can be considered agenticly engaged (15). By a consequence, the lack of discriminant validity between these two latent variables is not surprising.

### 4.1 Possibilities for future research

The development of holistic tools such as this scale that measure the different components of engagement will allow us to examine a number of pertinent and valuable questions regarding young people's participation in physical education. In order to conceptualize these possibilities, we turn to Dunkin and Biddle's model (38) for the study of classroom teaching which proposed four sets of variables which directly and indirectly influence student achievement. Using this model as a heuristic for research on engagement, we suggest that engagement could be considered as a process variable in some cases, but also as a product variable in others.

One of the hallmarks of the discourse on physical education is that it should be an inclusive subject so that all young people can experience the joy of movement and develop into physically literate individuals. However, evidence from a number of studies makes it clear that certain groups of students do not find physical education to be particularly inviting. What is less known is whether students with different characteristics entering physical education (motivation, skill, gender, etc.) engage at different levels. A foundational question with respect to the contextual variables might ask “do students with different characteristics entering physical education (motivation, skill, gender, goal orientation) engage at different levels?” Further, given that students have shown a preference for game-based physical education over a more fitness-focused program, we might ask “does the context of the lesson have an impact on student engagement?” Finally, given the advent of a number of instructional models in which students are expected to be active learners and are given the authority to make a number of decisions within lessons (39), we may be well served to ask “how do students engage in lessons when teachers adopt different instructional strategies?”

At the time of the Duncan and Biddle model, the examination of teaching styles was particularly nascent, while the idea of model-based practice was not part of the sport pedagogy lexicon. Teaching style here is used in reference to the spectrum of teaching styles first introduced by Mosston (40), and not to the motivational style of the teacher (a presage variable). From the research on teaching styles we know there are differential outcomes in terms of motivation (41) and changes in goal orientation (42). Nonetheless, it may well be interesting also to investigate differences in student engagement from a multidimensional approach (not simply behavioral) as these students experience these different instructional approaches.

## 5 Conclusion

In conclusion, the final model contained 18 of the original 21 items. Each of the four *a priori* latent variables was kept in the validation of the four-factor hypothesized model. Moderate to strong R2 between the latent variables and the items associated with the constructs. These findings indicate that the final 18-item model can be used to determine the self-reported levels of student agentic, cognitive, behavioral, and emotional engagement in physical education.

Some of the limitations of this research include purposeful sampling. Since only eighth and ninth graders were used, it may be difficult to generalize the results to other grade levels. Moreover, the survey's wording may need to be changed for research with younger students. Additionally, only 231 participants from two schools were included. A larger sample from more schools may have provided results that could have shown greater discriminant validity between agentic engagement and cognitive engagement.

Another limitation was the reduced number of items chosen for the cognitive subscale. Reeve and Tseng (15) included eight items in the cognitive subscale. The new EPES only included four items in the subscale. If all the original eight items were modified and included, the results might have shown improved discriminant validity between the cognitive and agentic engagement latent variables.

Considering the decades of research on student engagement and the new focus of student engagement in classroom tasks, the EPES can be used to complement many kinds of interventions in physical education to add different and meaningful aspects to a study. For example, a pre and post design for different curriculum models could be used to determine if student engagement changes due to the curriculum models chosen. Another example is how self-reported student engagement is impacted due to the teacher and student demographics. Additionally, future research could also pair individuals' self-reported results with the observation of the individual throughout an instructional unit in physical education.

## Data Availability

The raw data supporting the conclusions of this article will be made available by the authors, without undue reservation.
